# A Nomogram for Predicting Laparoscopic and Endoscopic Cooperative Surgery during the Endoscopic Resection of Subepithelial Tumors of the Upper Gastrointestinal Tract

**DOI:** 10.3390/diagnostics11112160

**Published:** 2021-11-22

**Authors:** Shun-Wen Hsiao, Mei-Wen Chen, Chia-Wei Yang, Kuo-Hua Lin, Yang-Yuan Chen, Chew-Teng Kor, Siou-Ping Huang, Hsu-Heng Yen

**Affiliations:** 1 Division of Gastroenterology, Changhua Christian Hospital, Changhua 500, Taiwan; 177099@cch.org.tw (S.-W.H.); 97601@cch.org.tw (C.-W.Y.); 27716@cch.org.tw (Y.-Y.C.); 182972@cch.org.tw (S.-P.H.); 2 Division of Gastroenterology, Yuanlin Christian Hospital, Changhua 500, Taiwan; 3 Department of Information Management, Chien-Kuo Technology University, Chunghua 500, Taiwan; 135442@cch.org.tw; 4 Department of Tumor Center, Changhua Christian Hospital, Changhua 500, Taiwan; 5 Department of General Surgery, Changhua Christian Hospital, Changhua 500, Taiwan; 120380@cch.org.tw; 6 Department of Hospitality Management, MingDao University, Changhua 500, Taiwan; 7 Big Data Center, Changhua Christian Hospital, Changhua 500, Taiwan; 179297@cch.org.tw; 8 Graduate Institute of Statistics and Information Science, National Changhua University of Education, Changhua 500, Taiwan; 9 General Education Center, Chienkuo Technology University, Changhua 500, Taiwan; 10 Department of Electrical Engineering, Chung Yuan Christian University, Taoyuan 320, Taiwan; 11 College of Medicine, National Chung Hsing University, Taichung 400, Taiwan

**Keywords:** subepithelial tumor, endoscopic resection, laparoscopy, GIST

## Abstract

Background: Considering the widespread use of esophagogastroduodenoscopy, the prevalence of upper gastrointestinal (GI) subepithelial tumors (SET) increases. For relatively safer removal of upper GI SETs, endoscopic submucosal dissection (ESD) has been developed as an alternative to surgery. This study aimed to analyze the outcome of endoscopic resection for SETs and develop a prediction model for the need for laparoscopic and endoscopic cooperative surgery (LECS) during the procedure. Method: We retrospectively analyzed 123 patients who underwent endoscopic resection for upper GI SETs between January 2012 and December 2020 at our institution. Intraoperatively, they underwent ESD or submucosal tunneling endoscopic resection (STER). Results: ESD and STER were performed in 107 and 16 patients, respectively. The median age was 55 years, and the average tumor size was 1.5 cm. En bloc resection was achieved in 114 patients (92.7%). The median follow-up duration was 242 days without recurrence. Perforation occurred in 47 patients (38.2%), and 30 patients (24.4%) underwent LECS. Most perforations occurred in the fundus. Through multivariable analysis, we built a nomogram that can predict LECS requirement according to tumor location, size, patient age, and sex. The prediction model exhibited good discrimination ability, with an area under the curve (AUC) of 0.893. Conclusions: Endoscopic resection is a noninvasive procedure for small upper-GI SETs. Most perforations can be successfully managed endoscopically. The prediction model for LECS requirement is useful in treatment planning.

## 1. Introduction

Subepithelial tumors (SETs) originate from muscularis mucosa, submucosa, or muscularis propria. In a Swedish population, the incidence of gastric SETs was reported to be 0.36% [1]. Considering the widespread use of esophagogastroduodenoscopy (EGD), the incidence increases. Recently in South Korea, the prevalence of gastric SETs was 1.7%, and increased to 3.0% in patients in their 60s [2]. Endoscopic ultrasound (EUS) can reveal the layer of origin, tumor size, and internal echotexture. However, EUS alone has been less accurate in diagnosing hypoechoic lesions with three to four layers [3]. Hence, histopathological diagnosis remains the gold standard. Current guidelines recommend surgical resection for patients with symptoms, tumor size > 20 mm, or high-risk features [4,5,6]. However, laparoscopic wedge resection may be difficult for tumors with a smaller size, protruding into the inner cavity of the stomach, or located in the lesser curvature or nearby esophagogastric junction [7]. Nevertheless, endoscopic submucosal dissection (ESD) has been considered as an effective and relatively safe method for removing superficial mucosal tumors, such as adenoma or early cancer, as well as SETs [8,9]. In this study, we aimed to retrospectively analyze the outcome of endoscopic resection for upper gastrointestinal (GI) SETs in our hospital, and develop a prediction model for the need of laparoscopic and endoscopic cooperative surgery (LECS) for the procedure.

## 2. Materials & Methods

### 2.1. Inclusion Criteria and Exclusion Criteria of the Study Population

We retrospectively analyzed the medical records of patients who received endoscopic treatment with intent of laparoscopic and endoscopic cooperative surgery (LECS) during the procedure for upper GI SETs between January 2012 and December 2020 at our institution. All patients received EUS evaluation or abdominal computed tomography to identify the tumor size, invasive depth, and differential diagnosis before endoscopic resection. Endoscopic tumor resection was performed in those with highly suspected gastrointestinal stromal tumors (GISTs) on EUS, tumor size >20 mm, or patient request. During the resection, patients underwent either ESD or submucosal tunneling endoscopic resection (STER), depending on the discretion of the endoscopist on the basis of the EGD/EUS image or computer tomography scan finding. Preoperatively, we consulted the general surgeon routinely for the possible need for LECS in cases where perforation occurred (with perforation, the endoscopist could not seal the site with clips because of full-thickness resection) or the tumor was deemed unsuitable for complete resection via endoscopy alone. Perforation of the procedure was defined as any visible defects of the muscle layer during endoscopic resection. Perforation with laparoscopic cooperative surgery was defined as the need for surgical intervention for uncontrolled situations during endoscopic resection. Delayed perforation was defined as the presence of peritoneal signs and imaging evidence of perforation from the surgical site following a successful endoscopic resection procedure. Delayed bleeding was defined the presence of a coffee ground substance from the nasogastric tube, vomiting with bloody or coffee ground substance, or passage of tarry stool following the endoscopic resection procedure. All the patients received general anesthesia and at the operation room. Patients who received endoscopic mucosal resection (EMR) or with the intent of thoracoscopic surgery were excluded.

### 2.2. Equipment and Procedure

The equipment used for ESD included Olympus endoscopes (GIF-H260Z and GIF-2TQ260M; Olympus Medical Systems Corp., Tokyo, Japan) with dual knife (KD-650l; Olympus Medical Systems Corp., Tokyo, Japan), IT Knife-2 (KD-612L; Olympus Medical Systems Corp., Tokyo, Japan), and an electrosurgical generator (ESG-100; Olympus Medical Systems Corp., Tokyo, Japan). The injection solution used to lift up the submucosal layer was composed of 10% glycerin and epinephrine (1:100,000). A CO_2_ insufflation system (UCR; Olympus Medical Systems Corp., Tokyo, Japan) was used to reduce patient discomfort during the ESD procedure. A typical ESD procedure was performed with mucosal incision with exposure of the subepithelial lesion followed by resection of the lesion in an attempt of R0 resection. A submucosal tunneling endoscopic resection (STER) was performed with mucosal incision 2–3 cm above the target lesion and passing the endoscope into the submucosal space for tumor resection. The mucosal defect was subsequently closed with multiple clips. In cases involving incidental perforation or uncontrolled bleeding that could not be resolved by endoscopic clips or hemostasis, general surgeons took over the procedure and completed the procedure with laparoscopic cooperative surgery (LECS).

### 2.3. Ethical Considerations

The study protocol conforms to the ethical guidelines of the 1975 Declaration of Helsinki. The Ethics Committee of Changhua Christian Hospital approved our study protocol (CCH IRB No.: 210202), and informed consent was waived, considering the study’s retrospective design.

### 2.4. Statistical Analysis

The extracted data were organized using Microsoft Excel and analyzed using MedCalc Statistical Software version 19.16 (MedCalc Software bvba, Ostend, Belgium; https://www.medcalc.org; accessed on 1 December 2020) or R software (R × 64 4.0.5, https://www.r-project.org/; accessed on 1 December 2020). The data was represented as mean SD or median (IQR) for continuous data and number (percentage %) for category data. Furthermore, *p* < 0.05 was considered statistically significant. The mean values of variables with non-normal distributions were compared using Mann–Whitney *U* test. The frequencies of categorical variables were compared using Pearson’s χ^2^ or Fisher’s exact test, as appropriate.

The data were randomly divided into training data and test data at 66% and 34%, respectively. To construct a prediction model for the need of LECS, we implemented a penalized logistic regression model and nomogram visualization in R software, with the use of clinical valuable features in the training dataset. The nomogram was constructed using the regression coefficient for each clinical valuable feature. The predictive accuracy and discriminative ability of the nomogram were determined by the area under the curve (AUC) and the calibration plot. For the calibration, we used the Hosmer–Lemeshow test, and compared the means of predicted salvage surgery with those of actual salvage surgery. The problem of overfitting was checked by three-fold cross-validation. Furthermore, the reliability of our nomogram was validated using independent test data.

## 3. Results

### 3.1. Clinical Features of the Study Population

This study analyzed 123 patients who underwent endoscopic resection for upper GI SETs between January 2012 and December 2020. Table 1 summarizes patients’ characteristics and histopathologic details. The mean age was 55 years. Males accounted for 49.6%. The average tumor size was 1.5 cm. Most tumors were located in the gastric body (*n* = 35, 28.4%), followed by the lower esophagus (*n* = 30, 24.3%), cardia (*n* = 21, 17%), fundus (*n* = 21, 17.0%), antrum (*n* = 14, 11.4%), and duodenum (*n* = 2, 1.6%). Most SETs arose from the muscularis propria (69.1%). Moreover, 56 (45.5%), 42 (34.1%) 8 (6.5%), and 3 (2.4%) patients had leiomyomas, GISTs, aberrant pancreases, and neuroendocrine tumors, respectively. En bloc resection was successfully performed in 114 (92.7%) patients. The median follow-up duration was 242 days without recurrence. Tumors located in the esophagus, gastric fundus, and gastric cardia were mostly leiomyoma (*n* = 25, 83.3%), GISTs (*n* = 17, 81.0%), and leiomyoma (*n* = 13, 61.9%), respectively (Table 2). GISTs were larger in size than leiomyomas or other tumors.

### 3.2. Analysis of Complication and Risk Factor Predictive for LECS

Out of the 123 patients who received endoscopic resection, 47 had perforation intraoperatively, and 30 of them required LECS (Table 3). Meanwhile, one patient had delayed perforation with abdominal pain and pneumoperitoneum symptoms and underwent laparoscopic surgery on the next day after ESD. LECS was most likely required in GIST cases (*p* = 0.0049). Lesions located at the gastric body or fundus had a higher rate of LECS than other lesion sites (32.6% vs. 1.5%, *p* < 0.001). Perforation was unrelated to tumor size. However, LECS was likely required for larger tumors.

### 3.3. Development and Validation of the Nomogram

**Figure 1:**
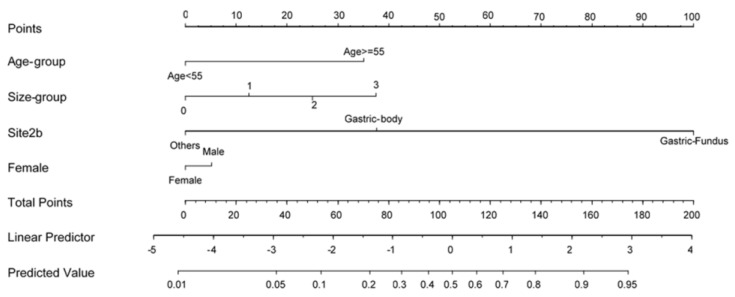
Nomogram of the prediction model.

**Figure 2:**
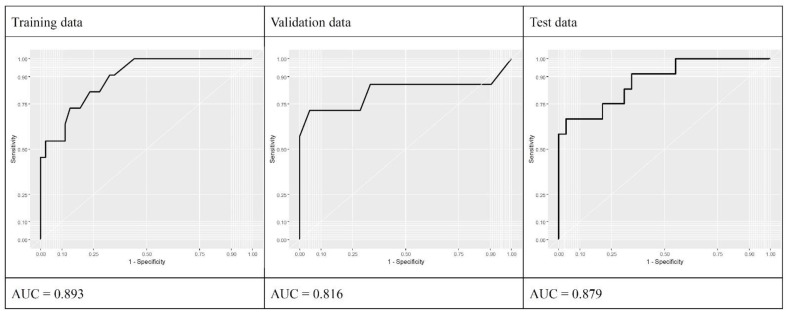
ROC analysis for predicting LECS requirement during endoscopic resection.

**Figure 3:**
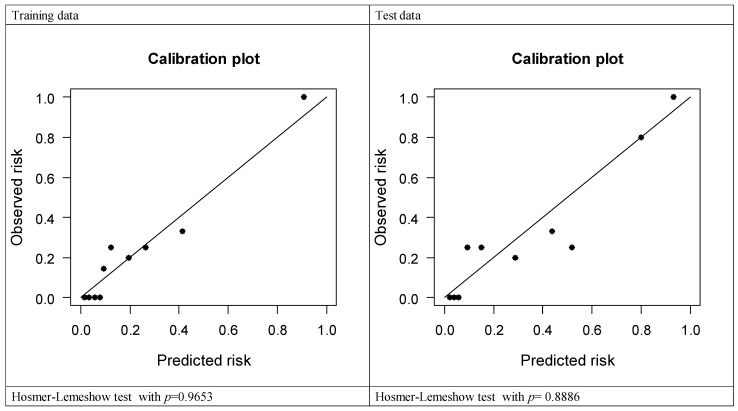
Calibration plot for predicting LECS requirement during endoscopic resection.

To develop a prediction model for LECS requirement during endoscopic resection, we analyzed the LECS-associated clinical factors (Table 4). A nomogram was constructed using variables such as age, sex, SET location, and tumor size (Table 5). Figure 1 illustrates the nomogram for each factor. The AUCs of the training, validation, and test data were 0.893, 0.816, and 0.879, respectively, implying a good discrimination ability (Figure 2). The nomogram was also well calibrated according to the calibration curve and Hosmer–Lemeshow test results (*p* = 0.9653 and 0.8886, respectively; Figure 3).

## 4. Discussion

As the amount of screening increases, the incidental identification of GISTs also increases, and this tumor type has become a common problem, especially for lesions <2 cm [5,10]. Owing to the low risk of disease progression, surveillance endoscopy alone is recommended; unfortunately, patient compliance is poor [11]. With the advancement of endoscopic resection techniques, endoscopic and/or laparoscopic management of these lesions has become possible. In this study, we evaluated a large case series in a single institution and developed a prediction model for LECS requirement during the endoscopic management of GI SETs [12,13,14].

GIST, which is a common gastric SET, has variations of malignant potential depending on tumor size and mitotic rate [15]. The most common location of malignant/potentially malignant SETs was the gastric body (78.1%), followed by the fundus (15.6%). In particular, GISTs were frequently found in the gastric body (25/31, 80.6%) and fundus (4/31, 12.9%) [16]. In our analysis, GISTs were mostly found at the gastric fundus. Meanwhile, leiomyomas were mostly located at the gastric cardia and esophagus.

The United States National Comprehensive Cancer Network and the Asian consensus guidelines suggested that surgical resection is strongly recommended when the tumor is ≥20 mm, is growing, or has malignancy signs, such as irregular margins, ulceration, bleeding, cystic change, necrosis, or heterogeneous echogenicity in endoscopy and/or EUS [4,17]. However, a small GIST size (<20 mm) has also malignancy potential. One study suggested 14 mm as a reasonable cutoff size for small GISTs because of significant tumor progression [18]. In our study, GISTs were highly mitotic when the tumor size was <2 cm (1/14 patients, 7.1%) and annual surveillance alone may be inadequate.

Furthermore, endoscopic resection was a safe alternative to surgical resection for removing upper GI SETs, especially for those with highly suspected GISTs clinically or with tumor size >2 cm [9]. However, perforation remains one of the major complications during ESD. With the improvement of endoscopic techniques, small perforations may be successfully sealed with clips intraoperatively. Large perforations may need laparoscopic surgery, with increased complication rates and prolonged hospitalization days [5,13,19,20,21]. The perforation rate during ESD for SET is reportedly 4–27.2% [9,22,23,24,25,26,27]. The perforation rate was significantly higher in the upper third of the stomach [9], particularly the fundus [23], and when the tumor invades the muscularis propria [25,27,28]. As we defined the perforation as any visible defects of the muscle layer fiber, to achieve a R0 resection, we encountered a higher rate of perforation in the present study.

In STER, which is a modified form of peroral endoscopic myotomy for esophageal achalasia [29], a submucosal tunnel is created for tumor resection. STER can resect SETs even with muscularis propria invasion and can well-preserve the clipped overlying mucosa [30,31]. A meta-analysis with 12 studies evaluated the safety of STER for SETs in 701 patients and found that the most common complications were subcutaneous emphysema and pneumomediastinum, with a perforation rate of 0.6% [32]. In our analysis, only 1 of 16 patients received salvage laparoscopic surgery after STER. Most tumors were located at the esophagus, gastric cardia, and gastric body. Tumors located at the gastric fundus were unsuitable for STER because of the sharp angle of the endoscopy approach and the difficulty in identifying tumor location at the submucosal tunnel.

In our institution, GISTs accounted for 81.0% of fundal SETs, and LECS was frequently demanded to avoid tumor seeding and local recurrence. Using a full-thickness resection device is potentially beneficial to solve such problems. Meanwhile, endoscopic full-thickness resection using novel devices, such as over-the-scope clip (OTSC) and overstitch endoscopic suturing system, is possible for SET resection without laparoscopic assistance [33,34,35]. However, these new devices are not all available in Taiwan, as well as in some other countries. Therefore, LECS is still required in our series, accounting for the high perforation/operation rate, especially in the gastric fundus. Furthermore, some retrospective studies reported a safe method for LECS for gastric SET that does not depend on the tumor location, such as the esophagogastric junction or pyloric ring [36,37,38].

As shown in our analysis, endoscopic resection is less invasive for small upper-GI SETs, and most perforations could be successfully managed by an endoscopic method. Meanwhile, LECS requires additional manpower during upper-GI SET management. Currently, we do not have a model that can help predict LECS requirement. In the present study, we developed a nomogram used for predicting the need for LECS. The nomogram is practical and highly accurate. For SETs that are highly likely to require LECS, using such a nomogram could help the endoscopist communicate with the surgeon and the patient regarding the treatment plan before the endoscopic resection, especially when the OTSC and overstitch endoscopic suturing systems are unavailable.

However, this study has some limitations [39,40]. First, this is a single-center study with a small sample size. Further large-scale studies are required to validate the usefulness of this nomogram for clinical use. Second, this study is retrospective and possibly has some selection biases. Not all upper-GI SETs were enrolled for endoscopic resection. Third, endoscopic resection, either ESD or STER, requires high-technology methods only experienced endoscopists could perform. In this study, two experienced endoscopist with at least 100 annual ESD experiences for colon neoplasms or upper-GI neoplasms performed the endoscopic resection. Thus, this nomogram could be useful for shared decision making with patients even with less experienced endoscopists preoperatively.

## 5. Conclusions

Endoscopic resection is noninvasive and safe for treating upper-GI SETs. The prediction model for LECS requirement is useful for endoscopists when communicating with the surgeon and the patient before endoscopic resection.

## Figures and Tables

**Table 1:** Characteristics of patients and their subepithelial tumors.

Characteristics		Number
Median age, years (Median, IQR)		55 (48–60.75)
Sex, *n* (%)		
	Male	61 (49.6%)
	Female	62 (50.4%)
Hospital day, days (Median, IQR)		6 (5–8)
Procedure time, minutes (Median, IQR)		60 (30–90)
Tumor location		
	Esophagus	30
	Gastric fundus	21
	Gastric cardia	21
	Gastric body	35
	Gastric antrum	14
	Duodenum	2
Tumor size, cm (Median, IQR)		1.5 (1–2.5)
Layer of tumor origin, *n* (%)		
	Submucosa	38 (30.9%)
	Muscularis propria	85 (69.1%)
Complications, *n* (%)		
	Perforation	47 (38.2%)
	Perforation with laparoscopic cooperative surgery	30 (24.4%)
	Delayed perforation	1 (0.8%)
	Delayed bleeding	1 (0.8%)
Pathology report, *n* (%)		
	Leiomyoma	56 (45.5%)
	GIST	42 (34.1%)
	Aberrant pancreas	8 (6.5%)
	Neuroendocrine tumor	3 (2.4%)
	Others *	14 (11.4%)
En bloc resection rate		114 (92.7%)
Follow-up days, day (Median, IQR)		242 (69–774)

* Others: glomangioma, elastofibroma, arteriovenous malformation, hemangioma, schwannoma, cavernous hemangioma, plexiform fibromyxoma, gastritis cystica profunda, lipoma. GIST, gastrointestinal stromal tumor; IQR, interquartile range.; Hospital day: Days from patient being admitted till patient being discharged. Follow-up days: Days from patient being discharged to the last clinical visit.

**Table 2:** Association of histopathology pattern and tumor location.

	Esophagus	Gastric Fundus	Gastric Cardia	Gastric Body	Gastric Antrum	DuodenuSm
GIST	1	17	7	14	3	0
Leiomyoma	25	2	13	16	0	0
Aberrant pancreas	0	0	0	1	6	1
Neuroendocrine tumor	0	0	0	2	0	1
Others	4	2	1	2	5	0

GIST, gastrointestinal stromal tumor.

**Table 3:** Comparison of patient features with or without LECS.

	Without LECS	With LECS	*p*-Value
Sex (M/F)	47/46	14/16	0.7134
Age, year (mean, SD)	51.79 (13.09)	58.16 (11.06)	0.0018
Size, cm (mean, SD)	1.61 (0.89)	2.20 (1.10)	0.0003
Site (E/Antrum/Body/Cardia/Fundus/D)	30/13/26/17/5/2	0/1/9/4/16/0	<0.0001
Pathology (GIST/Leiomyoma/Others)	22/49/22	20/7/3	0.0001
Resection method (ESD/STER)	78/15	29/1	0.0712

D, duodenum; E, esophagus; ESD, endoscopic submucosal dissection; F, female; GIST, gastrointestinal stromal tumor; LECS, laparoscopic and endoscopic cooperative surgery; M, male; SD, standard deviation; STER, submucosal tunneling endoscopic resection.

**Table 4:** Factors associated with LECS, according to univariable and multivariable analyses.

	Univariable Analysis			Multivariable Analysis		
	cOR (95% CI)	*p*-value	AUC	Coef.	adj. OR (95% CI)	*p*-value
Site			0.820			
Others	1				1	
Gastric body	3.57 (0.68, 18.65)	0.132		1.60	4.95 (0.78, 31.62)	0.091
Gastric fundus	100.8 (6.36, 1598.31)	0.001		4.25	70.32 (3.74, 1320.99)	0.005
Size (per 1 unit increase)	1.84 (0.94, 3.60)	0.076	0.658	0.53	1.70 (0.68, 4.27)	0.257
Age ≥ 55	3.91 (0.82, 18.56)	0.086	0.653	1.49	4.45 (0.61, 32.22)	0.140
Female	1.14 (0.31, 4.17)	0.845	0.517	−0.22	0.80 (0.15, 4.35)	0.800

AUC, area under the curve; CI, confidence interval; Coef., coefficient; cOR, crude odds ratio; LECS, laparoscopic and endoscopic cooperative surgery.

**Table 5:** Demographics of the training, cross-validation, and test data.

	Training Data	Validation Data	Test Data	*p*-Value
Sample size	54	28	41	
Age, years	54.89 ± 10.55	50.07 ± 12.57	53.56 ± 15.51	0.275
<55	23 (42.59%)	16 (57.14%)	20 (48.78%)	0.454
≥55	31 (57.41%)	12 (42.86%)	21 (51.22%)	
Sex				
Female	28 (51.85%)	15 (53.57%)	19 (46.34%)	0.807
Male	26 (48.15%)	13 (46.43%)	22 (53.66%)	
Site				
Gastric fundus	5 (9.26%)	3 (10.71%)	13 (31.71%)	0.041
Gastric body	18 (33.33%)	7 (25%)	10 (24.39%)	
Others	31 (57.41%)	18 (64.29%)	18 (43.9%)	
Size, cm	1.87 ± 1.04	1.67 ± 0.94	1.66 ± 0.92	0.516
<1	10 (18.52%)	6 (21.43%)	10 (24.39%)	0.811
≥1 to <2	23 (42.59%)	14 (50%)	18 (43.9%)	
≥2 to <3	10 (18.52%)	5 (17.86%)	9 (21.95%)	
3	11 (20.37%)	3 (10.71%)	4 (9.76%)	
LCES				
No	43 (79.63%)	21 (75%)	29 (70.73%)	0.604
Yes	11 (20.37%)	7 (25%)	12(29.27%)	

LCES: laparoscopic and endoscopic cooperative surgery.

## Data Availability

The datasets generated and/or analyzed during the current study are not publicly available, but these may be requested from the corresponding author, upon reasonable request.
